# Sentinel Lymph-Node Biopsy in Early-Stage Cervical Cancer: The 4-Year Follow-Up Results of the Senticol 2 Trial

**DOI:** 10.3389/fonc.2020.621518

**Published:** 2021-02-17

**Authors:** Guillaume Favre, Benedetta Guani, Vincent Balaya, Laurent Magaud, Fabrice Lecuru, Patrice Mathevet

**Affiliations:** ^1^ Department of Gynecology, University Hospital of Lausanne, Lausanne, Switzerland; ^2^ Faculty of Biology and Medicine, University of Lausanne, Lausanne, Switzerland; ^3^ Clinical Research and Epidemiology Department, Hospices Civils de Lyon, Lyon, France; ^4^ Faculty of Medicine, University of Lyon, Claude Bernard Lyon 1, Lyon, France; ^5^ Breast, Gynecology and Reconstructive Surgery Unit, Curie Institute, Paris, France; ^6^ Faculty of Medicine, University of Paris, Paris, France

**Keywords:** cervical cancer, sentinel lymph node, lymphadenectomy, survival, lymph node biopsy

## Abstract

**Introduction:**

Senticol 2 is a randomized multicenter trial in the treatment of early-stage cervical cancer patients. The aim of the Senticol 2 study was to compare the effect of sentinel-lymph-node biopsy (SLNB) to that of SLNB + pelvic lymphadenectomy (PLND), and to determine the postoperative lymphatic morbidity in the two groups. Here, we report a secondary objective of this study: the follow up.

**Material and Methods:**

In the Senticol 2 trial, patients underwent a laparoscopy with a sentinel-node-detection procedure and were randomized into two groups, namely: Group A, in which participants received SLNB, and Group B, in which participants received SLNB + PLND. Patients with an intra-operative macroscopically suspicious lymph node, were given a frozen-section evaluation and were randomized only if the results were negative. All of the patients received follow up with a clinical examination at 1, 3, and 6 months after surgery, and then every 3–4 months after that. The median follow up was 51 months (4 years and 3 months).

**Results:**

Disease-free survival after 4 years for the SLNB group and the SLNB + PLND group were 89.51% and 93.1% (*p* = 0.53), respectively. The only statistical factor associated with recurrence in the univariate analysis was the adjuvant radiotherapy. No other factors, including the age of the patients, histological type, tumor size, lymph vascular space invasion (LVSI), and positive nodal status, were significant in the univariate or multivariate analyses. The overall survival rates after 4 years in the SLNB and SLNB + PLND groups were 95.2% and 96% (*p* = 0.97), with five and four deaths, respectively. The univariate and multivariate analyses did not find any prognostic factors.

**Conclusions:**

This randomized study confirmed the results of the Senticol 1 study and supports the sentinel lymph node (SLN) technique as a safe technique for use in patients with early-stage cervical cancer treated with SLNB only. Disease-free survival after 4 years was similar in patients treated with SLN biopsy and patients who underwent a lymphadenectomy.

## Introduction

Cervical cancer results in over 300,000 deaths worldwide every year (1).

Advances in cervical-cancer screening have resulted in a lower incidence of cervical cancer but a higher incidence of early-stage disease diagnosis in developed countries (2, 3). One of the most important prognosis factors in the early stages is the pelvic lymph-node status.

It has been demonstrated that the lymph-node status directly impacts the 5-year survival rate of patients with International Federation of Gynecology and Obstetrics (FIGO 2009) Stage IA1 to IIB cancers (4).

According to the international guidelines for the treatment of early-stage cervical cancer, the gold-standard treatment includes pelvic-lymph-node dissection (PLND) in order to adapt the treatment to a potential lymphatic metastasis. In the Senticol 1 trial (5), we demonstrated the feasibility and safety of the sentinel lymph node (SLN) technique when used with bilateral detection. A review of the literature by Tax et al. showed 99% sensitivity and a 97–100% negative predictive value (NPV) for the bilaterally detected sentinel lymph node (SLN) technique (3).

A lymph-node metastasis is present in 27% of early cervical cancers, leading to a high rate of overtreatment with unnecessary PLND in three out of four patients (2, 3). Moreover, this lymphatic surgery is known to induce significant morbidity and to lead to a decreased quality of life (6).

A SLNB procedure can accurately detect metastases for several other diseases such as breast, penile, skin, and vulvar cancer. In 2015, the National Comprehensive Cancer Network (NCCN) Guidelines (7) stated that SLN mapping should be considered an option for PLND in cervical cancer (category 2B).

Furthermore, we showed previously that an SLN biopsy can decrease both early and long-term morbidity and can improve quality of life compared with a complete pelvic lymphadenectomy (6).

The aim of this study was to assess the disease-free survival and overall survival of early-stage cervical cancer patients included in the randomized controlled multicenter Senticol 2 study.

## Patients and Methods

### Patients

The Senticol 2 protocol (clinical trial #NCT01639820) was approved by an ethics committee (Comité de Protection des Personnes Sud-Est IV, decision A08-223) and all of the patients provided written informed consent before inclusion.

We performed a randomized controlled trial from December 2008 to November 2011. A total of 25 centers were included, all surgical team were experimented (>20 cases). The number of cases per center and the name of the surgeon is described in **Supplementary Table 2** in the **Appendix**.

Patients were followed for a minimum of 3 years after their inclusion in the study.

The inclusion criteria were as follows: Women aged 18 or older diagnosed with cervical carcinoma of FIGO 2009 stage IA1 with LVSI, to IIA1, including any histological subtype (except neuroendocrine carcinoma). The patients were eligible for laparoscopy and written informed consent was obtained from all of the patients.

The exclusion criteria were as follows: Pregnant women, stage IB (by downstaging), evolving or recurrent cervical cancer, other cancer diagnosed during treatment, history of pelvic node surgery, or severe allergy or contraindication to radioactive tracer or Patent Blue.

### Methods

All patients received injections of the radioactive tracer colloidal rhenium sulfide labeled with technetium (99mTc; Nanocis^®^, CIS Bio International) on the day of (60 MBq; short protocol) or the day before the surgery (120 MBq; long protocol), after which 2 mL of vital dye (Patent Blue^®^, Laboratoire Guerbet) diluted with 2 mL of water was injected into the cervix at the 3, 6, 9, and 12 o’clock positions.

In addition, a pre-operative lymphoscintigraphy was performed in order to detect SLNs during surgery, especially in unexpected locations.

The patients underwent a laparoscopy using a sentinel-node-detection procedure and were randomized into the following two groups: Group A, which received sentinel lymph node biopsy (SLNB), and Group B, which received SLNB + PLND.

The patients with intra-operative macroscopically suspicious sentinel or eventually non sentinel lymph nodes (NSLNs) received a frozen-section evaluation and were randomized only if the results were negative.

All of the SLNs were analyzed using the histological ultrastaging method (200 µm sections) and were stained with hematoxylin eosin saffron (HES) or hematoxylin phloxine saffron (HPS). An additional immunohistochemistry (IHC) analysis with a pan-cytokeratine antibody was performed in case of a negative SLN.

For the definite node-negative patients, we proceeded with an additional surgery—either a radical hysterectomy or radical trachelectomy (an extrafascial hysterectomy or simple trachelectomy were performed for tumors <2cm without lymph vascular space invasion (LVSI)). Adjuvant treatments were defined following the final histology.

For the definite node-positive patients, we proceeded with an additional treatment using chemo-radiotherapy after first considering a laparoscopic para-aortic lymphadenectomy.

All of the patients received clinical examination follow ups 1, 3, and 6 months after surgery, and then every 3–4 months afterwards. The median follow-up duration was 51 months.

### Statistical Methods

Disease-free survival rates were estimated using the Kaplan Meier method. The survival curves were compared with a Log-rank test (unilateral test with a 5% significant threshold). Recurrence-free survival was defined as the time from surgery to disease recurrence or death due to any cause. A second analysis regarding patients with no evidence of recurrence or death was done at the date of the last follow-up. A multivariate analysis was performed including factors with *p* < 0.15. The multivariate analysis of the recurrence-free survival was performed using a Cox’s proportional hazard model. All of the analyses were performed on an intention-to-treat basis.

## Results

### Characteristics of Patients

Between December 2008 and November 2011, 267 patients participated in Senticol 2. Of these, 61 patients were excluded—2 (3.3%) had not had a lymphoscintigraphy before surgery, 12 (19.7%) had an incomplete SLNB procedure, 11 (18%) had an absence of SLN detection, 21 (34.4%) had a unilateral detection of SLN, and 15 (24.6%) had positive SLN on the frozen sections.

A total of 206 patients were randomized, with 105 patients assigned to Group A (SLNB) and 101 to Group B (SLNB + PLND).

The baseline characteristics of the patients at inclusion are summarized in **Table 1**. The median ages were 42.2 and 41.7 years, respectively. Most patients (88.4%) had FIGO stage IB disease. Histological subtypes represented were mainly epidermoid carcinoma (64.8% and 72.3%, respectively). There was a nonsignificant difference between the two groups in terms of the size of the tumors (median sizes were 19 and 15 mm, respectively).

**Table 1:** Patient characteristics at baseline.

	SLNB *n* = 105	PLND *n* = 101	*p* value
Age (median)	42.2	41.7	0.81
BMI (median)	22.7	22.6	0.92
History of abdominal surgery (n (%))	69 (65.7)	70 (69.3)	0.66
PS score 3 2 1	88 (95.7) 4 (4.3) 0	88 (96.7) 2 (2.2) 1 (1.1)	0.19
History of pregnancy (n (%))	85 (81)	87 (86.1)	0.43
Menopausal status (n (%))	29 (28.2)	30 (30)	
FIGO 2009 stage at inclusion I A1 with LVSI I A2 I B1 II A1	7 (6.7) 5 (4.8) 90 (85.7) 3 (2.9)	2 (2.2) 6 (6.0) 91 (91.0) 1 (1.0)	0.29
Histology (n (%)) Epidermoid carcinoma Adenocarcinoma Adenosquamous carcinoma Other	68 (64.8) 33 (31.4) 2 (1.9) 2 (1.9)	73 (72.3) 24 (23.8) 2 (2.0) 2 (2.0)	0.68
Preoperative conization (n (%)) Presence of LVSI in the biopsy (n (%))	68 (64.8) 19 (27.9)	63 (62.4) 16 (25.4)	0.52 0.84
Surgical approach of radical hysterectomy: Laparotomy Laparoscopy Vaginal-assisted laparoscopy	6 42 41	4 40 38	0.87

SLNB, sentinel lymph node biopsy; PLND, pelvic lymph node dissection; BMI, body mass index; PS, performance status; LVSI, lymph vascular space invasion.

All patients received injections of the 96 technetium and 2 mL of vital dye (Patent Blue^®^) diluted with 2 mL of water injected into the cervix at the 3, 6, 9, and 12 o’clock positions. We had no cases with discrepancy between the lymph-nodes marked with patent blue and lymph-nodes marked with technecium. During the SLN procedure, there was no difference in sentinel mapping between the two groups (**Supplementary Table 1** in the **Appendix**). The main location for the sentinel node was ilio-obturator and external iliac area (85.8%). The second main location was the common iliac area (9.6%) of SLN.

The surgical approaches were similar in the two groups (*p* = n.s.), with a radical hysterectomy rate of 78.7% and 80.5%, a radical trachelectomy rate of 16.9% and 13.4%, and a simple hysterectomy and trachelectomy rate of 4.5% and 6.1%, respectively.

The final histological analysis (including ultrastaging) of the SLNs indicated 12 (11.4%) patients with a positive node in the SLNB group (three with macro-metastasis, three with micro-metastasis, and six with isolated tumor cells) and 9 (8.9%) patients with a positive node in the SLNB + PLND group (three with macro-metastasis, four with micro-metastasis, and two with isolated tumor cells) (*p* = n.s.). In this group there were 9 positive SLN and 1 positive NSLN in a patient with a positive SLN also. The correspondence between SLN and NSLN was 100%.

The rate of postoperative adjuvant therapy was similar in the two groups (**Table 2**), including brachytherapy (32 in the SLNB group and 27 in the SLNB + PLND group), radiotherapy (13 and 16, respectively), and chemotherapy (9 and 11, respectively). Nine patients from the SLNB group underwent a secondary lymph-node dissection in raison of positive SLN on the final pathology. This secondary dissection was performed according to the protocols of the different centers. One patient had only a pelvic lymphadenectomy, 6 patients had pelvic and para-aortic dissection, and 2 patients had only para-aortic dissection. We observed one case of positive para-aortic node in a patient with micrometastasis in the SLN, and one patient with 2 pelvic positive nodes on the secondary pelvic lymphadenectomy after metastatic SLN.

**Table 2:** Post-operative adjuvant therapy.

Additional treatment	SLNB	SLNB + PLND	*p* value
Brachytherapy Radiotherapy Chemotherapy	32 (30.8%) 13 (12.5%) 9 (8.7%)	27 (26.7%) 16 (15.8%) 11 (10.9%)	0.54 0.55 0.64

SLNB, sentinel-lymph-node biopsy; PLND, pelvic lymph-node dissection.

### Survival Outcomes

The mean follow-up duration was 50 months (3–89 months), with a median of 51 months (4 years and 3 months).

**Figure 1:**
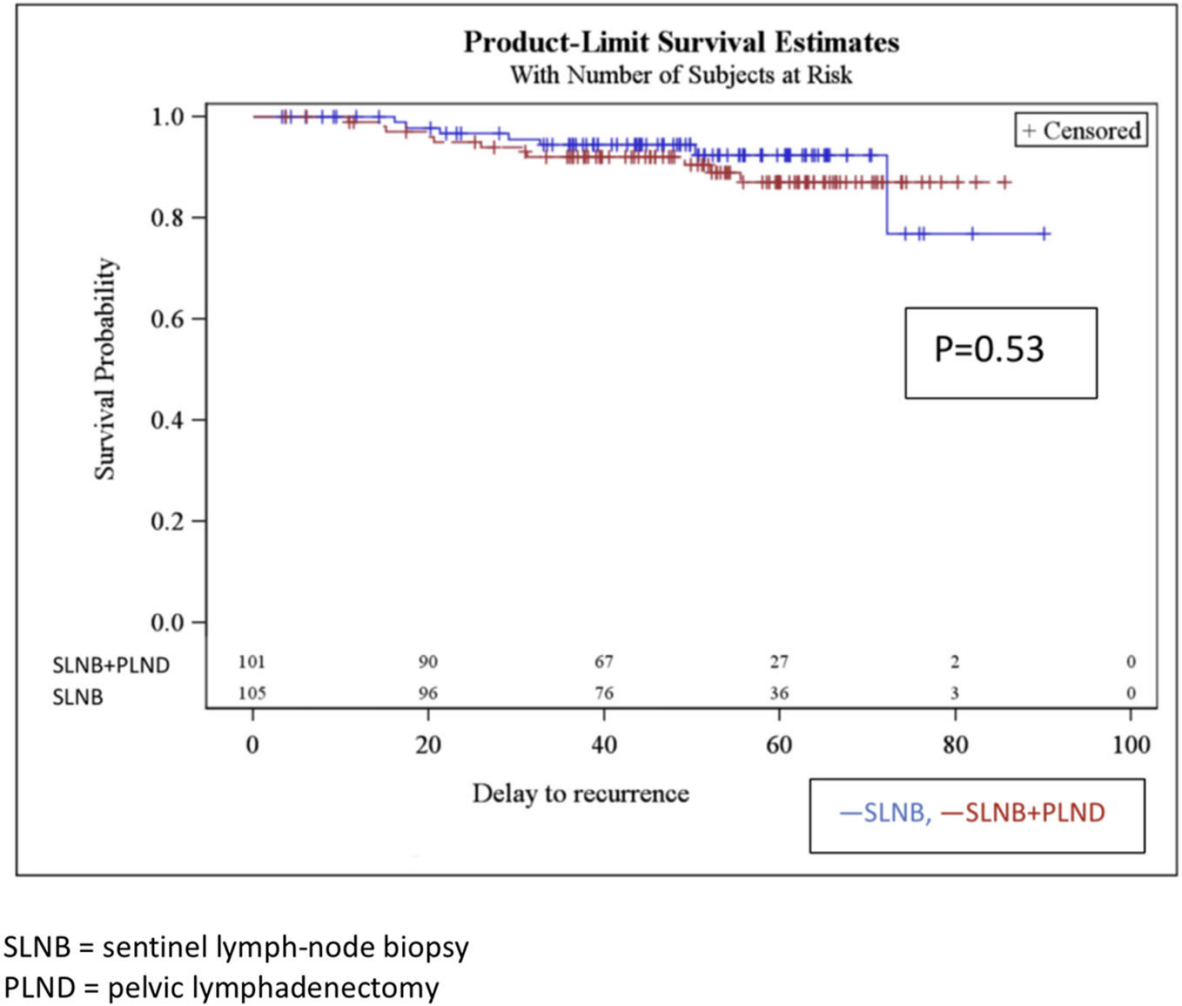
Disease-free survival rate at 4 years.

Disease-free survival after 4 years in the SLNB and SLNB + PLND groups was 89.5% and 93.1% (*p* 161 = 0.53), respectively. There was no significant difference between the two groups. There were 11 recurrences in the SLNB group and 7 in the SLNB + PLND group (**Figure 1**). The most statistically significant factor identified in the univariate analysis was radiotherapy. In fact, we observed 11 recurrences in the patients treated with adjuvant radiotherapy (15.5%). Other factors that influenced disease-free survival after 4 years in the univariate and multivariate analyses included the age of the patients, histological type, tumor size, presence of LVSI, positive nodal status, surgical approach, and adjuvant treatment. However, a trend was observed, depending on the case, for LVSI. In the multivariate analysis, both of these covariates (LVSI and adjuvant radiotherapy) were significant (**Table 3**). The type of recurrences is described in **Table 4**; there were 2 lymphatic recurrences: one pelvic in the SLNB group and one para-aortic (associated with liver metastasis) in the SLNB + PLND group.

**Table 3:** Univariate and multivariate analyses of the association between disease-free survival and risk factors.

Risk factors	Total number	Recurrences	*P* value (univariate analysis)	*P* value (multivariate analysis)
**Histological type**			0.97	
Squamous	135	12 (8.9%)		
Adenocarcinoma	56	5 (8.9%)		
Adenosquamous	6	1 (16.7%)		
Other	9	0		
**Tumor size**			0.92	
<2 cm	28	2 (7.2%)		
>2 cm	176	16 (9%)		
**Lymph node involvement**			0.87	
Yes	21	2 (9.5%)		
No	185	16 (8.6%)		
**Type of surgery**			0.97	
Simple hysterectomy	5	0		
Simple trachelectomy	4	0		
Radical hysterectomy	171	16 (9.3%)		
Radical trachelectomy	26	2 (7.7%)		
**Type of radical hysterectomy**			0.13	
Type B	158	10 (6.3%)		
Type C	13	3 (23%)		
**Lymph-vascular space invasion**			0.09	0.16
Pos	48	7 (14.9%)		
Neg	159	11 (6.9%)		
**Adjuvant Radiotherapy**			**0.02**	0.25
Yes	71	11 (15.5%)		
No	135	7 (5.2%)		

**Table 4:** Type of recurrences.

Group	Recurrence	Time of recurrence	Death
SLNB	Lungs	18 months	Yes
SLNB	Lungs	14 months	No
SLNB	Parametrium	15 months	Yes
SLNB	Lungs	49 months	No
SLNB	Pelvic	19 months	Yes
SLNB	Inguinal	49 months	No
SLNB	Lymphatic (right iliac)	52 months	No
SLNB	Vaginal	9 months	Yes
SLNB	Liver	26 months	Yes
SLNB	Peritoneum	30 months	No
SLNB	Vaginal	30 months	No
SLNB + PLND	Carcinosis and lungs	50 months	Yes
SLNB + PLND	Parametrial	72 months	No
SLNB + PLND	Lungs	17 months	Yes
SLNB + PLND	Lungs and liver	15 months	Yes
SLNB + PLND	Lymphatic (para-aortic) and liver	20 months	Yes
SLNB + PLND	Vaginal	28 months	No
SLNB + PLND	Peritoneum	31 months	No

SLNB, sentinel lymph node biopsy; SLNB+PLND, sentinel lymph node biopsy + pelvic lymphadenectomy.

During the follow-up period, nine deaths were observed (four in the SLNB + PLND group and five in the SLNB group). The overall survival (OS) for the patients who received SLNB was 95.2, and 96% for those who received SLNB + PLND (*p* = 0.97). In the univariate analysis, none of the risk-factor covariates that were analyzed were statistically significant for any of the clinical events at a *p* = 0.05 level. However, a trend could be observed, depending on the case, for the covariates—namely adjuvant radiotherapy and LVSI. When we retained covariates with a *p*-value of <0.15 in the univariate analysis in a multivariate model, none were statistically significant (**Table 5**).

**Table 5:** Univariate and multivariate analyses of the association between risk factors and overall survival.

Risk factors	Total number	Death	P stat (univariate analysis)	P stat (multivariate analysis)
**Histological type**			0.93	
Squamous	135	7 (5.2%)		
Adenocarcinoma	56	2 (3.6%)		
Adenosquamous	6	0		
Other	9	0		
**Tumor size**			0.25	
<2 cm	28	2 (7.2%)		
>2 cm	176	7 (3.9%)		
**Lymph-node involvement**			0.88	
Yes	21	1 (4.8%)		
No	185	8 (4.3%)		
**Type of surgery**			1	
Simple hysterectomy	5	0		
Simple trachelectomy	4	0		
Radical hysterectomy	171	9 (5.3%)		
Radical trachelectomy	26	0		
**Type of radical hysterectomy**				
Type B	158	9 (5.7%)		
Type C	13	0		
**Lymph-vascular space invasion**			0.12	0.22
Pos	48	4 (8.5%)		
Neg	159	5 (3.1%)		
**Adjuvant radiotherapy**			0.07	0.11
Yes	71	6 (8.5%)		
No	135	3 (2.2%)		

## Discussion

Full pelvic lymph-node dissection is associated with early and long-term morbidity. Limited dissection of SLNB is associated with lower morbidity and a better quality of life (6).

Moreover, the SLN procedure has been intensely assessed, and has shown safe and relevant results as follows: considering bilateral SLN detection with histological ultrastaging, a 99–100% sensitivity with a 97–100% NPV was demonstrated (3). While this technique has been used in tumors up to 4 cm in size, the best detection rates and mapping results are in tumors less than 2 cm (7). The ultrastaging techniques, combining serial sectioning and IHC, improved the rate of nodal metastasis detection and revealed that 8.1% of apparently node-negative patients were classified as node-positive. Low-volume metastasis (micrometastasis and isolated tumor cells) is usually only detected *via* ultrastaging. Its clinical significance is currently being debated. The FIGO 2018 (8) classification considers only macro- and micro-metastases to be significant lymph-node metastases; the presence of isolated tumor cells does not change the stage. Our recent study, presented at the ASCO 2020 Congress (9) and recently published (10), demonstrated that the sentinel-node technique is reliable even from the point of view of low-volume metastasis in NSLN. The limitation of the ultrastaging technique is that it cannot be performed intraoperatively as it is too cumbersome and time-consuming. Considering the convenience of a single-step approach in early stage cervical cancer, the one-step nucleic acid amplification (OSNA) assay has recently been investigated in several tumor types, including cervical cancer patients (11). More important results are expected on the study of this technique in cervical cancer in order to be able to draw conclusions about its reliability.

In our study, the results showed that for women with early-stage cervical cancer from FIGO (2009) IA1 to IIA1, the SLN procedure alone did not result in a significant disease recurrence compared to complete pelvic lymph-node dissection. In addition, no significant difference was found in the overall survival between the two groups.

Considering that a great majority of recurrences normally appear within 3 years after treatment, SLNB seems to be a safe and efficient alternative to lymphadenectomy, and should be proposed to every patient as a routine protocol for early-stage cervical cancer in cancer centers where surgeons are familiar with this technique and follow the correct SLN protocol. It should still be considered that the study was not designed to compare the survival and the risk of recurrence between the two groups (SLNB and SLNB + PLND). This subgroup of patients with early-stage cervical cancer indeed had a good survival with a low recurrence rate. In conclusion, too few events were observed during long-term follow up (4 years) to allow for a tangible analysis of the risk factors for survival or disease-free survival.

However, this was the first randomized controlled prospective study evaluating the follow up of SLNB alone in comparison with SLNB + PLND, and it confirmed the Senticol 1 results.

Considering the other factors associated with risk of recurrence, only the adjuvant radiotherapy was statistically significant (*p* = 0.02). This result can be explained by the fact that the patients who presented major risk criteria (according to the Seidlis criteria) (12) were treated with radiotherapy. In the multivariate analysis, no factors were significant for either the risk of recurrence or for survival.

Recently, the LACC study by Pedro Ramirez (13) demonstrated the superiority of open surgery for better survival and disease-free survival in the treatment of early-stage cervical cancer. Our study was designed before Ramirez’s study, and the vast majority of our patients were operated on *via* laparoscopy. Open surgery was performed only in the case of complications during laparoscopic surgery. It is interesting that in our study, we found a survival and disease-free survival similar to that of the open-surgery group in Ramirez’s study. This comparison is limited, as our study was designed for laparoscopic treatment and there were a large number of cases where the colpotomy was performed vaginally with protective measures. A vaginal colpotomy may protect from the risk of tumor dissemination and recurrence (14).

## Conclusions

SLN biopsy was found to be a safe technique allowing for accurate nodal staging in early cervical cancer.

Furthermore, this surgery led to less morbidity and to a clearly improved quality of life for patients. Given that disease recurrence after 4 years was similar in patients who underwent an SLN procedure, this study strongly supports the extension of this surgical approach to all clinically node-negative patients (cN0) affected by early cervical cancer.

A strong and influential study called Senticol 3 (15) and SentiX (16) is under way in order to confirm the equivalent survival in SLNB and SLNB + PLND patients.

## Data Availability Statement

The raw data supporting the conclusions of this article will be made available by the authors, without undue reservation.

## Ethics Statement

The studies involving human participants were reviewed and approved by Comité de Protection des Personnes Sud-Est IV, decision A08-223. The patients/participants provided their written informed consent to participate in this study.

## Author Contributions

Conceptualization, GF, VB, BG, FL, and PM. Methodology, LM. Software, LM. Validation, VB, FL, BG, and PM. Investigation, FL and PM. Resources, BG, PM, and FL. Data curation, GF, BG, PM, and FL. Writing—original draft preparation, GF and BG. Writing—review and editing, GF, VB, FL, BG, and PM. Visualization, GF, VB, FL, BG, and PM. Supervision, FL and PM. Funding acquisition, PM. All of the authors have read and agreed to the published version of the manuscript. All authors contributed to the article and approved the submitted version.

## Funding

The Senticol 2 study was supported by the French National Cancer Institute (STIC 2008 and 2012). The study sponsor had no role in the design or conduct of the study, interpretation of data, or the writing of the report.

## Conflict of Interest

The authors declare that the research was conducted in the absence of any commercial or financial relationships that could be construed as a potential conflict of interest.
