# Supplementary material for: Sentinel Lymph-Node Biopsy in Early-Stage Cervical Cancer: The 4-Year Follow-Up Results of the Senticol 2 Trial
Source: Front Oncol. 2021 Feb 17;10:621518. doi: 10.3389/fonc.2020.621518 (PMC7927597; doi:10.3389/fonc.2020.621518)
Supplement: Supplementary file 1 [file DataSheet_1.docx]

|  | **Appendix** |
| --- | --- |
|  |  |
|  | **Table 1.** Anatomical topography of sentinel lymph nodes. |

| Topography of SLN | SLNB | SLNB + PLND |
| --- | --- | --- |
| - Ilio-obturator/external iliac - Common iliac - Parameter - Promontory - Para-aortic - Other | 355 (86.6%)  35 (8.5%)  3 (0.7%)  8 (2.0%)  5 (1.2%)  4 (1.0%) | 306 (85.0%)  38 (10.6%)  5 (1.4%)  6 (1.7%)  1 (0.3%)   1. (1.1%) |

SLN = sentinel-lymph node; SLNB = sentinel lymph-node biopsy; PLND = pelvic lymph-node dissection

**Table 2.** Centers and surgeons participating in Senticol 2 trial:

| **Center** | **N. of randomized patients** | **Surgeon** |
| --- | --- | --- |
| Institut Gustave Roussy Villejuif | 49 | P. Morice |
| HFME Lyon | 26 | P. Mathevet |
| Cancer Center Bordeaux | 21 | E. Stoeckle |
| Cancer Center Toulouse | 15 | D. Querleu |
| Institut Curie Paris | 13 | V. Fourchotte |
| HEGP Paris | 12 | F. Lecuru |
| Cancer Center Rouen | 9 | M. Baron |
| University Hospital Rennes | 7 | J. Leveque |
| University Hospital Reims | 7 | O. Graesslin |
| Tenon University Hospital Paris | 5 | E. Darai |
| Mulhouse Hospital | 5 | B. Ott |
| Centre Jean Perrin Clermont Ferrand | 5 | C. Pomel |
| Mougins Private Hospital | 5 | D. Lanvin |
| University Hospital Tours | 4 | H. Marret |
| University Hosp Clermont Ferrand | 4 | G. Mage |
| University Hospital Bordeaux | 4 | V. Conri |
| University Hospital Strasbourg | 3 | JJ. Baldauf |
| University Hospital Marseille | 2 | G. Houvenaeghel |
| University Hospital Dijon | 2 | S. Douvier |
| Centre A.Lacassagne Nice | 2 | Y. Fouché |
| Centre O.Lambret Lille | 2 | E. Leblanc |
| University Hospital Angers | 1 | P. Descamps |
| Cancer Center Nantes | 1 | J.M. Classe |
| CHLS Lyon | 1 | F. Golfier |
| University Hospital Lariboisière Paris | 1 | E. Barranger |
|  | **206** |  |

**Senticol 2 group:**

E. Stoeckle, Institut Bergonié, Bordeaux, France

V. Fourchotte, Institut Curie, Paris, France

D Querleu, Institut Claudius Régaud, Toulouse, France

M. Baron, Centre Henri Becquerel, Rouen, France

B. Ott, Hôpital du Hasenrain, Mulhouse, France

E. Daraï, Hôpital de Tenon, Paris, France

J. Lévèque, Hôpital Sud Anne de Bretagne, Rennes, France

D. Lanvin, Clinique de l’Espérance, Mougins, France

C. Pomel, Centre Jean Perrin, Clermont Ferrand, France

H. Marret, Hôpital Bretonneau, Tours, France

E. Leblanc, Centre Oscar Lambret, Lille, France

G. Houvenaeghel, Institut Paoli-Calmettes, Marseille, France

P. Rouanet, Centre Val d’Aurelle, Montpellier, France

P. Descamps, Centre Hospitalier Universitaire, Angers, France

G. Mage, Centre Hospitalier Régional Universitaire, Clermont Ferrand, France

O. Graesslin, Institut Mère Enfant, Reims, France

JJ. Baldauf, Hôpital de Haute Pierre, Strasbourg, France

JM. Classe, Centre René Gauducheau, Nantes, France

D. Raudrant, Centre Hospitalier Lyon Sud, Lyon, France

V. Conri, Hôpital Pellegrin, Bordeaux, France

S. Douvier, Centre Hospitalier Universitaire, Dijon, France

Y. Delpech, Hôpital Lariboisière, Paris, France

P. Leguévaque, Hôpital Rangueil, Toulouse, France

Y. Fouché, Centre Antoine Lacassagne, Nice, France

L. Boulanger, Hôpital Jeanne de Flandre, Lille, France

A.S. Bats, Hôpital Européen Georges Pompidou, Paris, France

C. Uzan, Institut Gustave Roussy, Villejuif, France

F. Bouttitie, Service d’informatique, CHLS, Lyon, France
